# Metasurface-Based Imagers Enabled Arbitrary Optical Convolution Processing

**DOI:** 10.1038/s41377-022-00792-x

**Published:** 2022-04-19

**Authors:** Minsu Park, Yeonsang Park

**Affiliations:** 1 Department of Physics, Chungnam National University, Daejeon, 34134 Korea; 2 Institute of Quantum Systems, Chungnam National University, Daejeon, 34134 Korea

**Keywords:** Imaging and sensing, Metamaterials

## Abstract

Using meta-imagers composed of a meta-lens and a complex-amplitude meta-modulator, all-optical convolutional processing that arbitrarily reshapes the point spread function of an optical system can now be implemented.

## Abstract (web summary)

Using meta-imagers, all-optical convolutional processing that arbitrarily reshapes the point spread function of an optical system can now be implemented.

## Graphical abstract

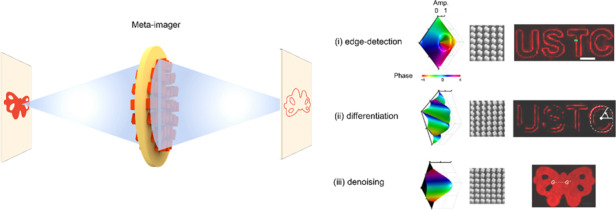

Image processing is a technology that transforms an image into the desired form by applying numerous convolution operations to digital image data, and it is increasingly becoming a core technology in science and engineering^1–3^. In particular, due to the recent remarkable development of the convolutional neural network (CNN)^4,5^, the importance and applications of image processing are increasing day by day. CNN is a mathematical and computational model that gives weighted outputs by connecting numerous numbers of layers (neurons, or nodes) to use a convolution operator as a single layer, and shows excellent performance in image recognition, image classification, image segmentation, etc. However, digital image processing using electronic devices has weaknesses such as limited computational speed due to low response of electrons, heat generation, and a lot of energy consumption. Therefore, many studies on optical signal processing or optical computation that processes light information in large-scale and real-time have been conducted^6,7^.

Generally, there are two main methods of optical signal processing^8^. One is a method using Fourier optics (Fourier spatial filtering)^9,10^, and the other is a method using Green’s function^11,12^. In Fourie optics, optical components such as a lens or a spatial filter (modulation mask) can modulate optical signal as a function of its frequency components. The Fourier method processes optical data by modulating the desired frequency component using a lens and a spatial filter (modulation mask) and reconstructing it with an inverse Fourier transformation. Therefore, The Fourier method has a disadvantage in that it is not suitable for a miniaturized integrated system because it uses the existing complex and bulky optical elements. Additionally, arbitrary convolution processing requires not only the modulation of light amplitude but also the modulation of the light phase. Since traditional optical elements can manipulate only the amplitude of light, the Fourier method has the disadvantage of not being able to implement complex-amplitude modulation for arbitrary convolution operation. Green’s function method executes optical signal processing by modulating the transmission (or reflection) of nanophotonic structure with various approaches. Although Green’s function method has the advantage of miniaturizing the whole system because the designed structure works as a single device, it has many constraints in designing and fabricating devices to operate versatile functions depending on various needs.

To realize an optical processor with compactness and versatile functions, a lot of nanophotonic systems using metamaterials^13^, metasurfaces^14^, photonic crystals^15^, surface plasmon-based devices^16^, topological photonics^17^, etc. have been reported nowadays. However, a structure capable of complex-amplitude modulation (controlling the phase and intensity of light simultaneously) required for all-arbitrary optical convolution processing has not been published yet.

Now, writing in *Light: Science & Applications*, Weiwei Fu et al reported a doublet meta-imager consisting of a meta-lens for image magnification and a meta-modulator that controls both the phase and amplitude of light as shown in Fig. 1, opening up new possibilities for metasurface-based CNN processors^18^. Any imaging system can be described as a convolution operation between the optical field of an object and the point spread function (PSF) of the optical elements that make up the optical system. Weiwei Fu et al introduced a rigorous mathematical analysis to calculate the optical pulse response of a modified imaging system by linking a direct relation between complex-amplitude modulation of meta-modulator and arbitrary convolution operation. Based on the introduced analysis, Weiwei Fu et al proposed and experimentally demonstrated a compact meta-imager structure for versatile convolution operations such as image edge-detection, edge-enhancement, differentiation, and denoising. In particular, compared to the existing image processing that adjusts only the phase or amplitude of light, optical image processing using the complex-amplitude meta-imager that adjusts both the phase and amplitude enabled denoising image processing that cannot be achieved with existing methods. Additionally, it was experimentally confirmed that the detection accuracy was improved due to the complex-amplitude modulation. Since the proposed meta-imager consists of a metalens for image magnification and a meta-modulator for complex-amplitude modulation, precise alignment between two structures is required during the fabrication. Nevertheless, because the meta-imager by Weiwei Fu et al enables all-optical convolution operation and high integration, it is expected to be used as a device for the CNN node, and this work will open a new path for the emergence of metasurface-based CNN artificial intelligence (AI) apparatus.

**Fig. 1:**
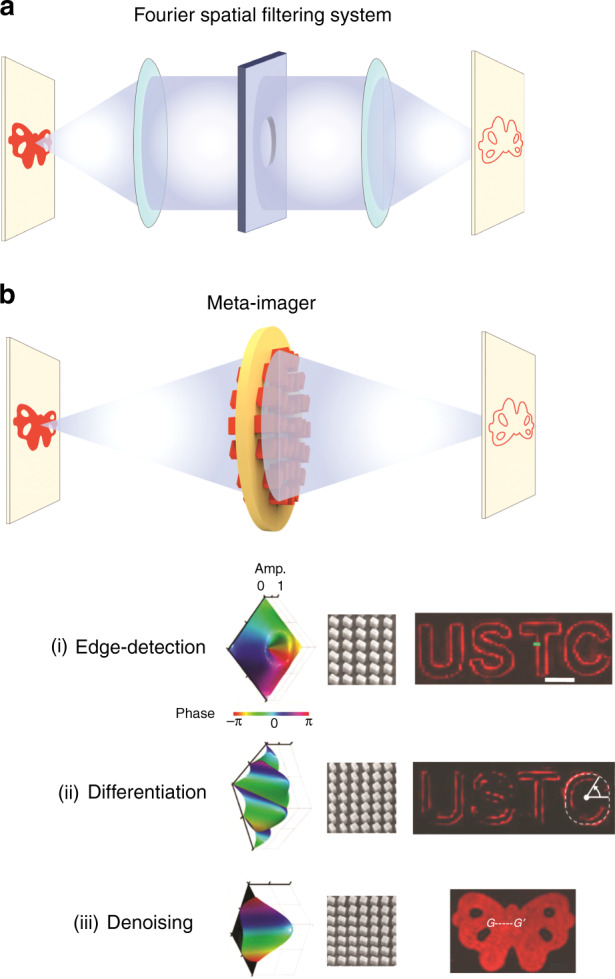
Schematics of optical image processing system. **a** Schematic of a Fourier spatial filtering system. **b** Schematic of a meta-imager, (i–iii) edge-detection, differentiation, and denoising with a meta-imager, (From left to right) phase and amplitude contours of a meta-modulator, SEM images of the fabricated meta-modulator, and measured images (scalebar: 50 μm) reproduced from ref. ^18^
